# Validation of multiplex immunofluorescence for use in analysis of tumour infiltrating lymphocytes

**DOI:** 10.1186/2051-1426-3-S2-P411

**Published:** 2015-11-04

**Authors:** Henry Galetta, James Mansfield, Byers Richard, Kenneth K Oguejiofor

**Affiliations:** 1 The Medical School, The University of Manchester, Manchester, UK; 2 PerkinElmer, Inc., Hopkinton, MA, USA; 3 Department of Histopathology, Manchester Royal Infirmary, Central Manchester University Hospitals NHS Trust, Manchester, UK; 4 Institute of Cancer Sciences, The University of Manchester, Manchester, UK

## Background

Multiplexed immunohistochemistry (IHC) has the potential to improve conventional IHC staining allowing for analysis of multiple cell phenotypes while maintaining spatial context. Automated multispectral image analysis and computer-based cell recognition make the process more attainable, but stringent validation of multiplex IHC is still required. Pertinently, multiplex IHC allows for the characterisation and enumeration of immune cell densities in the tumour micro-environment, of particular importance for analysis of tumour-infiltrating immune cells which require multiple co-localised markers for their identification. However, issues of antibody blocking, cross-reactivity and masking have caused concern that the results of multiplex staining may not accurately reflect those of single-plex staining. The Opal workflow from PerkinElmer uses a heat-induced epitope retrieval step between each antibody detected which aims to obviate these potential problems and enable single-species antibody use. In this study we systematically validated multiplex staining for a range of immune cell markers against single-plex staining for each marker to determine the accuracy of the multiplex method.

## Methods

Validation of multiplex IHC was undertaken using a multi-tissue TMA stained in multiplex for CD3, CD4, CD56 and CD20 in a 4-marker validation and for CD3, CD4, CD8, CD20 and FOXP3 in a 5-marker validation. The TMA was composed of 72 cores including normal lung, pancreas, breast, prostate and stomach and malignant prostate, lung and colon. Spearman correlations measured agreement of immune cell populations with those of singly stained serial sections on a per-core basis. Single-stain/single-stain comparisons of corresponding immune cell populations provided baseline variation of immune cells in subsequent sections.

## Results

All validation comparisons showed a highly significant correlation (P < 0.0001) with strong correlation was observed between cores for the majority of multiplex stains. Specifically, for the 4-plex experiment, a high degree of correlation was observed for CD3, CD8 and CD20 with R^2^ of 0.835, 0.950 and 0.870 respectively (P= < 0.001); CD56 showed a lower degree of correlation between the multiplex and single stains with an R^2^ of 0.584 (P < 0.0001). For the 5-plex experiment, a high degree of correlation was observed for high degree of correlation for CD3, CD8 and CD20 with R^2^ of 0.87, 0.95 and 0.87 respectively (P < 0.0001). FOXP3 showed a poor correlation of 0.74 and CD4 showed a poor correlation of 0.58 (P < 0.0001).

## Conclusion

Multiplex IHC is comparable to single stain IHC preserving precious samples and reagents while enhancing the information gained.

